# Structure of the fimbrial protein Mfa4 from *Porphyromonas gingivalis* in its precursor form: implications for a donor-strand complementation mechanism

**DOI:** 10.1038/srep22945

**Published:** 2016-03-14

**Authors:** Patrik Kloppsteck, Michael Hall, Yoshiaki Hasegawa, Karina Persson

**Affiliations:** 1 Department of Chemistry, Umeå University, Umeå, SE-901 87, Sweden; 2 Department of Microbiology, School of Dentistry, Aichi Gakuin University, 1-100 Kusumoto-cho, Chikusa-ku, Nagoya, Aichi 464-8650, Japan

## Abstract

Gingivitis and periodontitis are chronic inflammatory diseases that can lead to tooth loss. One of the causes of these diseases is the Gram-negative *Porphyromonas gingivalis*. This periodontal pathogen is dependent on two fimbriae, FimA and Mfa1, for binding to dental biofilm, salivary proteins, and host cells. These fimbriae are composed of five proteins each, but the fimbriae assembly mechanism and ligands are unknown. Here we reveal the crystal structure of the precursor form of Mfa4, one of the accessory proteins of the Mfa1 fimbria. Mfa4 consists of two β-sandwich domains and the first part of the structure forms two well-defined β-strands that run over both domains. This N-terminal region is cleaved by gingipains, a family of proteolytic enzymes that encompass arginine- and lysine-specific proteases. Cleavage of the N-terminal region generates the mature form of the protein. Our structural data allow us to propose that the new N-terminus of the mature protein may function as a donor strand in the polymerization of *P. gingivalis* fimbriae.

Gingivitis and periodontitis are chronic inflammatory diseases that can lead to tooth loss. One of the causes of these diseases is the Gram-negative, black-pigmented *Porphyromonas gingivalis*^1^, a bacterium also associated with the onset of cardiovascular disease, rheumatoid arthritis, diabetes and pancreatic cancer^2^,^3^,^4^. *P. gingivalis* has a diverse arsenal of virulence factors, including arginine- and lysine gingipains (cysteine proteases), lipopolysaccharides, and fimbriae^5^. The bacterium uses a specific secretion system, type IX, for delivering virulence factors like the gingipains and at least one accessory fimbrial protein to the outer membrane^6^. Fimbriae are hair-like, polymerized protein structures expressed on the surfaces of bacteria allowing them to cling to various surfaces. *P. gingivalis* expresses two fimbrial types, FimA and Mfa1. Both are crucial for the infectivity and survival of the bacteria as they attach to oral streptococci and other microorganisms in the dental biofilm, salivary proteins, and host cells^7^. The two fimbrial types are genetically distinct from each other and expressed from separate gene clusters^8^. Despite low sequence similarity, they have a similar architecture and are built up from five proteins each; FimA from proteins FimA-E, and Mfa1 from Mfa1-5 (Fig. 1a). In both fimbriae, the first proteins encoded by the gene cluster, FimA and Mfa1 respectively, polymerize into the fibrillar shaft, i.e., the main building block. The second proteins, FimB and Mfa2, are important regulators of fimbrial lengths, but are not found in the actual fimbrial structure^9^,^10^. The next proteins, FimC-E and Mfa3-5, compose the fimbrial tip. They presumably have important functions as adhesins, but their ligands are unknown^11^,^12^.

The FimA or Mfa1 assembly mechanisms are not known either. Most of what is known about fimbrial polymerization is based on *Escherichia coli* type-1 fimbria. These fimbriae are polymerized via a chaperone-usher mechanism^13^,^14^, in which a six-stranded, incomplete Ig-like fold of a fimbrial protein is completed by a donor strand from a chaperone, preventing premature aggregation in the bacterial cytoplasm. Upon polymerization, assisted by the membrane-bound usher, the chaperone β-strand is displaced by a donor strand from the next fimbrial subunit.

Although *P. gingivalis*, like *E. coli*, is Gram-negative, the assembly of the *P. gingivalis* fimbriae is poorly understood and no ushers or fimbrial chaperones have been reported. However several of the fimbrial proteins are processed in two steps, first by signal peptidase II, that removes the signal peptide, and secondly by the arginine specific gingipain (RGP) that trims the proteins into the forms found in the mature fimbria^15^. Analysis of native FimA and Mfa1 fimbriae, purified from *P. gingivalis* ACTCC 33277, shows that several of the fimbrial proteins are trimmed to their mature forms by RGP, undergoing cleavage after arginines located at position 43–54^11^. Hereafter we refer to the region between the signal peptide and this RGP cleavage site as the “N-terminal extension” (Fig. 1b).

*P. gingivalis* is an oral pathogen that affects most humans in industrialized and developing countries. We are convinced that there is a need to investigate the structure and function of its virulence factors and to generate platforms for drug targets that extend beyond the paradigm of *E. coli*. Fimbriae are important in virulence, but little is known about the maturation events leading to fimbrial polymerization and the roles of the individual components for *P. gingivalis*. This lack of knowledge is partly due to the lack of structural information; hence, we present the 1.9 Å resolution crystal structure of the *P. gingivalis* fimbrial tip protein Mfa4 in its precursor form. The structure allows us to propose a function for the N-terminal extension. Although it is not a part of the mature fimbriae, it forms an integrated part of the Mfa4 crystal structure. Furthermore, we constructed point mutants of the RGP cleavage site, Arg53, to investigate the effect of the N-terminal extension on the maturation and fimbrial incorporation of Mfa4. It may function as a putative donor strand for fimbrial polymerization.

## Results

### Construct design, crystallization and analysis of crystal contents

Mfa4 is predicted to localize to the bacterial outer membrane^12^ before it is integrated into the fimbria. Indeed, the LipoP server^16^ predicts a lipidation signal peptide including the first 18 residues (confidence score 11.4). Mfa4 has a serine (instead of an aspartic acid) at the +2 position (Ser20), which indicates that it localizes to the outer membrane by the lipoprotein outer-membrane localization (lol) pathway^17^. Immediately following the signal peptide is the N-terminal extension, a stretch of 35 amino acids not part of the mature fimbriae and with unknown function. Two constructs were made, representing the mature form, Mfa4_54–333_, and the precursor form, Mfa4_26–333_.

Diffraction quality crystals of selenomethionine-labelled Mfa4 (Mfa4_26–333_) were obtained from protein that was treated by *in situ* proteolysis with α-chymotrypsin immediately before crystallization screening. The crystals belong to space group *P*2_1_2_1_2_1_ with cell dimensions a = 54.68, b = 84.54 and c = 138.36 Å, containing two molecules in the asymmetric unit.

### Overall structure of Mfa4

**Figure 2:**
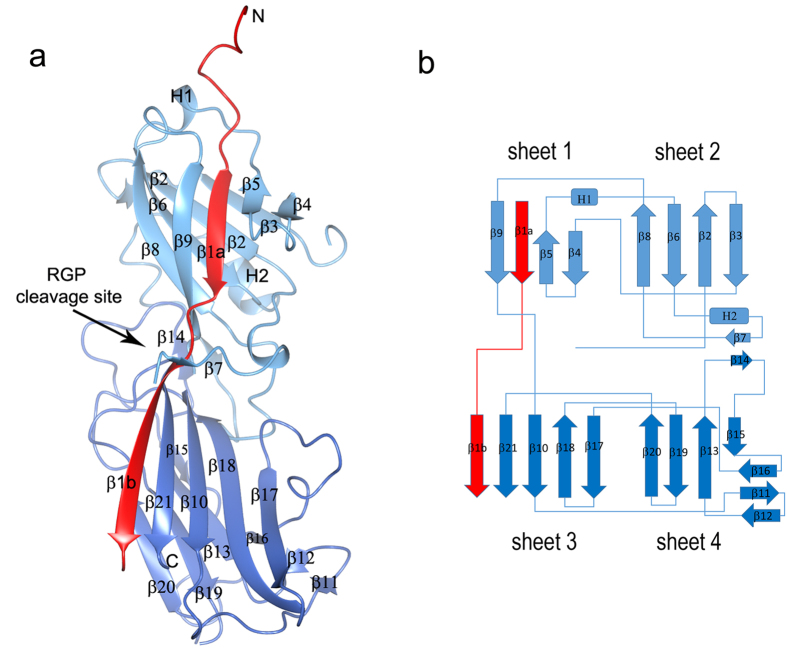
Overall structure of Mfa4. (**a**) Schematic representation of Mfa4 structure in which the N-domain is depicted in light blue and the C-domain in dark blue. The N-terminal extension is coloured red. The RGP cleavage site is indicated. (**b**) Topology diagram of Mfa4. β-strands are represented as arrows, helices as rectangles and loops as lines. Colours are the same as in (**a**).

The protein contains two domains (Figs 2 and S1). The N-terminal domain is comprised of a β-sandwich consisting of two four-stranded β-sheets packed against each other (sheets 1 and 2). The β-sandwich is flanked by two helices and a coiled region that packs against sheet 2. The C-terminal domain is also a β-sandwich, consisting of one five-stranded and one four-stranded sheet (sheets 3 and 4, respectively). In addition, the C-terminal β-sandwich is flanked by a small, three-stranded β-sheet. Several long loops and a short β-strand from each domain pack against sheet 4. The electron density is of high quality and easily interpretable except for the loop β3β4 (residues 89–93) which is likely to have been opened by α-chymotrypsin at Phe88. Similarly, the loop β19β20 (residues 298–308) is disordered and probably cleaved at Phe308. These regions have not been included in the final model, which was refined to an R value of 18.35% (R_free_ = 22.95%).

**Figure 3:**
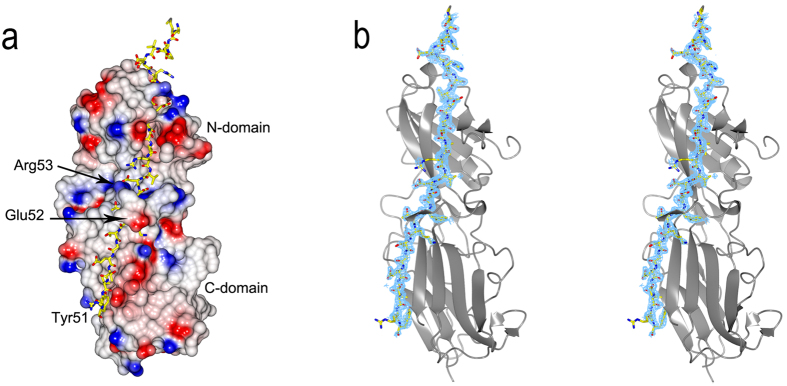
The N-terminal extension of Mfa4. (**a**) The N-terminal extension shown as a stick model where the rest of Mfa4 is presented as an electrostatic surface. (**b**) The Mfa4 N-terminal extension is modelled in an Fo-Fc simulated-annealing omit-difference map contoured at 2.5 sigma and shown in stereo.

An electrostatic surface of Mfa4 was computed using CCP4MG^18^ and displays a structure where no significant positive, negative or hydrophobic areas are present (Fig. 3a). Refinement statistics are presented in Table 1.

### The N-terminal extension

During fimbrial assembly, the first 18 Mfa4 residues are removed by signal peptidase II, leaving the protein linked to the membrane via Cys19. The remaining polypeptide is trimmed by RGP and cleaved after Arg53, leaving the mature form of Mfa4 to start at Asn54^11^. However, this construct, Mfa4_54–333_, expressed as an insoluble protein, so, a longer form, Mfa4_26–333_, was used for structural studies. Diffraction quality crystals were obtained after *in situ* proteolysis using α-chymotrypsin, resulting in a cleavage between Tyr51 and Glu52, closely mimicking the RGP cleavage that is performed *in vivo* after Arg53. The N-terminal extension (residues Glu26-Tyr51), which is not included in the native fimbria, forms two consecutive β-strands (β1a and β1b) that run through both the N- and C-domains of the protein (sheets 1 and 3). Strikingly, the N-domain β1a strand has an integrated position in the sheet, located between β5 and the last strand of the domain, β9. In the C-domain, the successive part of the strand, β1b, is located on the edge of the sheet, parallel to the final, C-terminal strand, β21. Intriguingly, after the break in the protein chain, Tyr51 and the following residue, Glu52, are 20 Å apart. The continuation of the chain forms a short β-strand, one helical turn, and a few coils after which the chain forms the β2-strand located in sheet 2 of the β-sandwich. The electron density is unambiguous for this part of the protein (Fig. 3b).

### Structural comparison with pg0181 from *P. gingivalis* W83

During preparation of this manuscript the Joint Center for Structural Genomics deposited the analogous protein from the *P. gingivalis* strain W83, PG_0181, in the Protein Data Bank (pdb 4rdb). As expected, the structures are practically identical (rmds 1.0 on 303 aligned Cα residues), except for the loops cleaved by α-chymotrypsin in the Mfa4 structure; the loop β1β2 which is flexible and only partly modelled in 4rdb; and the loops β3β4 and β19β20, which are fully modelled in 4rdb (Figure S2).

### Structural comparison

**Figure 4:**
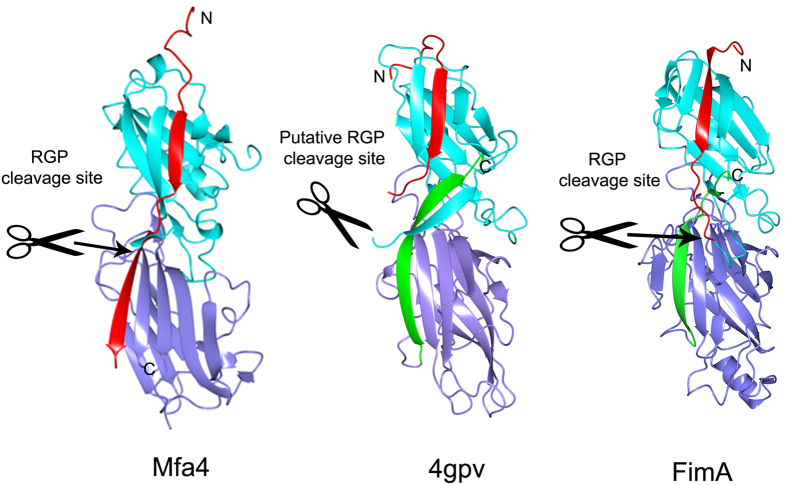
Structural features of Mfa4 and related proteins. Mfa4, the putative cell adhesin protein from *B. eggerthii* (4gpv), and FimA from *P. gingivalis* W83 are compared. Their N-terminal extensions, N-, and C-domains are colored red, cyan and purple, respectively. The C-terminal strands of 4gpv and FimA are shown in green. The RGP cleavage site is marked with scissors.

A search for structural homologs of Mfa4 was performed using DALI^19^. The search identified several structures with Z-scores up to 15.9 (Table 2). The top hit (Z-score 15.9 and rmsd 3.1 on 221 aligned Cα residues) corresponds to a putative cell adhesion protein from *Bacteroides eggerthii*, (pdb 4gpv) followed by three fimbrial proteins from *Parabacteroides distasonis* (pdb 3r4r, 4jg5 and 3liu). These four proteins are expressed by organisms commonly found in the gastrointestinal flora, they all belong to the Bacteroidetes phylum, and they are annotated as putative fimbrial proteins. Further down on the list is FimA from the *P. gingivalis* strain W83 (pdb 4q98) with a Z-score of 13.3 and an rmsd of 3.1 on 207 aligned Cα residues. These structures are all solved by the Joint Center for Structural Genomics and unpublished. The proteins exhibit similar overall topology, with some exceptions, especially regarding the position of the last strands, which is correlated to the length of the proteins (Fig. 4).

A shared detail of these structurally related proteins is that they all have a conserved cysteine immediately after the signal peptide; most of them are located at positions 19–21, which is predicted to be lipidated and linked to the outer membrane. In the two *P. gingivalis* fimbriae several of the involved proteins— FimA, Mfa1, Mfa3 and Mfa4—have mature forms, all starting after an arginine at position 43–53^11^. In *P. gingivalis*, this maturation process is performed by RGP. Assuming that the fimbrial maturation events are conserved among *Parabacteroides* and *Bacteroidetes* we expected the proteins in Table 2 to exist as lipidated precursors before maturation^20^,^21^ and to have a protease recognition site in their N-terminal extension, exposed on the β1β2 loop (Fig. 4).

**Figure 5:**
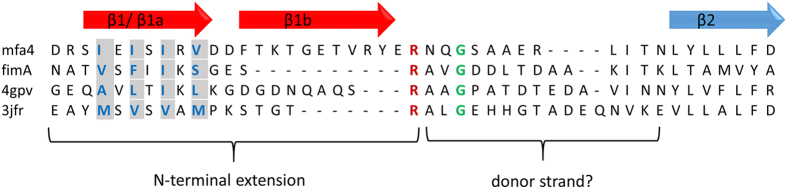
Structure-based sequence alignment of the N-terminal region. The N-terminal sequences of Mfa4 and structurally related proteins with an exposed arginine in the β1β2-loop. The conserved arginine is depicted in red. Hydrophobic amino acids in the β1/β1a strand are depicted in blue and highlighted in grey. Horizontal arrows indicate β-strands (β1b is only present in Mfa4). The location of the N-terminal extension is indicated.

The proteins obtained from the DALI search were crystallized as full-length proteins with their N-terminal extensions intact; the constructs have not been made to mimic any putative mature forms of the fimbrial proteins. Mfa4 was also expressed with its N-terminal extension; however, crystallization in the presence of α-chymotrypsin cut the polypeptide chain. Interestingly, this generated a difference in topology—in Mfa4 the cleaved N-terminal extension resulted in an elongated strand (β1a-β1b) that stretches over two domains. In the other, uncleaved structures found in DALI, the N-terminal extension forms a strand, β1, integrated in sheet 1. The β1 strand (or β1a in Mfa4) is tightly bound in all proteins listed in Table 2. In Mfa4, the β1a strand is mainly anchored via Ile33, Ile35, Ile37, Val39, residues accommodated by hydrophobic pockets (Fig. 5). The proteins in Table 2 have hydrophobic residues at the three first corresponding positions whereas the sequence variation is larger in the fourth position. This is also reflected by the more open binding pocket for the fourth residue. The β1b strand, that can only be formed in Mfa4 due to the chymotrypsin opening (mimicking RGP) of the β1β2 loop, runs parallel to the C-terminal strand, β21, and is positioned on the edge of the sheet. Due to the low number of hydrophobic residues and its positon at the edge of the sheet, β1b has few interactions except for the main chain hydrogen bonds to β21. The residues equivalent to β1b in the other, uncleaved proteins, form a long flexible loop between β1 and β2 in sheet 2 (β1β2-loop), which differs both in length and sequence. The crystal structure of FimA from *P. gingivalis* W83 represents the full-length protein with the RGP site intact (Arg45), located on the extended β1β2-loop following the first β-strand. Similarly, 4jg5 and 3up6 each expose a lysine in their analogous, fully modelled loops. In the other structures in Table 2, the β1β2-loop is partly flexible and the putative protease sites are not modelled. However, based on their sequences, they all have an exposed arginine or lysine in the loop, which prompts for recognition by RGP or alternatively, lysine-specific gingipain (KGP).

In the position comparable to the Mfa4 β1b-strand in the C-domain, all the other structures in Table 2 have their C-terminal strand. However, it runs in the direction opposite to that in Mfa4 β1b. In several of the structures, for instance 4gpv, the long C-terminal strand is extended into the β1β2-loop.

### Processing of Mfa4 by gingipains

**Figure 6:**
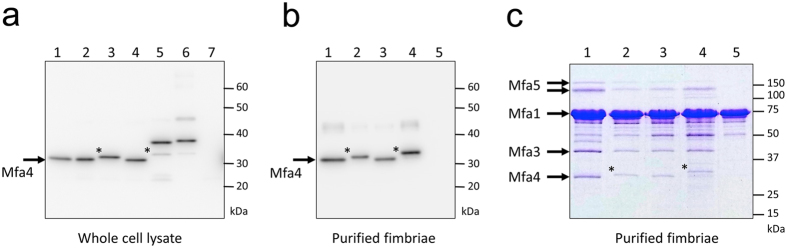
Mfa4 expression and immunoblot analysis of the point mutants. (**a**) Immunoblot analysis of Mfa4 protein. The whole-cell lysates were separated on SDS-PAGE and probed with an Mfa4 antibody by immunoblotting. Lanes: 1, JI-1; 2, JI-1 Mfa4WT; 3, JI-1 R53A; 4, JI-1 R53K; 5, JI-1 R50A/R53A; 6, KDP112(Δ*rgpA*/*B*); 7, FMFA4 (Δ*mfa4*, negative control)^40^ (**b**) Immunoblot analysis of Mfa4 from purified Mfa1 fimbriae. Lanes: 1 JI-1; 2, JI-1 R53A; 3, JI-1R53K; 4, JI-1 R50A/R53A; 5, FMFA4. (**C**) SDS–PAGE of the purified Mfa1 fimbriae. Lanes: 1, JI-1; 2, JI-1 R53A; 3, JI-1 R53K; 4, JI-1 R50A/R53A; 5, FMFA4. Asterisks indicate the Mfa4 bands analysed by N-terminal sequencing.

To further investigate the mechanism of Mfa4 processing by gingipains, point mutations were constructed in the *fimA* null mutant *P. gingivalis* strain JI-1 (Mfa4WT, Mfa4R53A and Mfa4R53K). Whole cell lysates separated by SDS-PAGE were subjected to immunoblotting with antisera raised against Mfa4 (Fig. 6a). The anti-Mfa4 antibody detected a band of 31 kDa in the parent strain of JI-1 and in JI-1 Mfa4WT, corresponding to the processed mature form of the protein. However, in JI-1 Mfa4R53A, there was a slight shift of the corresponding band to a higher molecular mass (32 kDa band). A similar increase in the apparent molecular weight of purified Mfa1 fimbriae from JI-1 Mfa4R53A was detected by SDS-PAGE (Fig. 6b). As it was probable that the observed mass increase was a result of upstream cleavage of the Mfa4 precursor protein, the band was excised and subjected to N-terminal sequencing. The Mfa4 band from the purified JI-1 Mfa4R53A fimbriae was found to start with the residue Tyr51 (sequence YEANQGSAAE). The prior residue was arginine, suggesting that the Mfa4R53A protein was instead processed at Arg50 by RGP to yield the fimbria associated forms.

Interestingly, the Mfa4 band in JI-1 Mfa4R53K, where Arg53 was replaced with lysine, showed the same molecular mass as for JI-1 and JI-1 Mfa4WT (Fig. 6b). This suggested that Lys53, instead of Arg53, could be used for processing of Mfa4, although presumably by KGP rather than RGP. This was further confirmed when the double mutant Mfa4R50A/R53A was constructed. The whole-cell lysate from this double mutant showed both a strong 38 kDa and a weaker 33 kDa band on SDS-PAGE (Fig. 6a). We assume that the 38 kDa band in the whole-cell lysate is the unprocessed form, i.e., the lipid precursor, and that the 33 kDa a processed mature form. The Mfa4 from purified fimbria only showed the processed 33 kDa band (Fig. 6b). N-terminal sequencing of this 33 kDa band revealed that the protein was processed at Lys44 (sequence TGETVAYEAN), again showing that KGP can rescue the trimming mechanism when suitable RGP recognition sites are missing and that only mature forms of Mfa4 can be incorporated into the fimbria.

In parallel we analyzed whole-cell lysate from an RGP-deficient mutant KDP112^22^ which had the same-sized Mfa4 bands as in the Mfa4R50A/R53A mutant (Fig. 6a).

It is important to note that no cleavage appears to have occurred downstream the N-terminal extension, at Arg61, which is also located on the surface exposed β1β2-loop, just following the helical turn. Furthermore, the purification of the fimbriae demonstrated that Mfa3, 4, and 5 co-purified with Mfa1 in all strains (Fig. 6c), implying that the mutations do not lead to any disruptions in the fimbrial assembly. On the basis of the intensity of the bands corresponding to the different Mfa proteins, it appears that the Mfa3, 4, and 5 subunits exist at an approximate 1:1:1 ratio in the purified fimbriae.

## Discussion

The *P. gingivalis* fimbria Mfa1 is a multifunctional surface appendage—which together with the second fimbria FimA—is important for the initial attachment of the bacteria in the oral biofilm as well as the bacteria’s ability to spread to non-oral sites in the body. The crystal structure of the *P. gingivalis* fimbrial accessory protein Mfa4 in its precursor form was obtained after *in situ* proteolysis using α–chymotrypsin. This generated a cleavage in the N-terminal extension closely mimicking that which is performed *in vivo* by RGP during the native assembly of the fimbria.

We found that the N-terminal extension, β1, is an integrated part of the first β-sheet of the N-terminal domain, running parallel to the last strand of the sheet. This is analogous with the strand arrangement observed when a β-strand from a chaperone completes the Ig-like fold of an *E. coli* or *Yersinia pestis* fimbrial subunit, thereby preventing premature polymerization and aggregation^23^,^24^. Upon polymerization of the *E. coli* type-1 fimbria, the chaperone β-strand is displaced by the N-terminal strand of another fimbrial protein using a zip-in–zip-out mechanism. This results in a β-strand that runs in the opposite direction compared to the chaperone donor strand^25^. This elaborate mechanism joins the fimbrial subunits into a growing fimbria.

In *P. gingivalis* no fimbrial chaperones have yet been identified, but Mfa4 can be recombinantly expressed in soluble form without substantial aggregation when the N-terminal extension is present. The crystal structures of Mfa4 and the related Bacteroidetes proteins indicate that the presence of the N-terminal extension is important for maintaining a soluble protein. Although the underlying mechanisms are still unknown, the proper cleavage and rearrangement of the N-terminal extensions are crucial for fimbria elongation *in vivo*.

**Figure 7:**
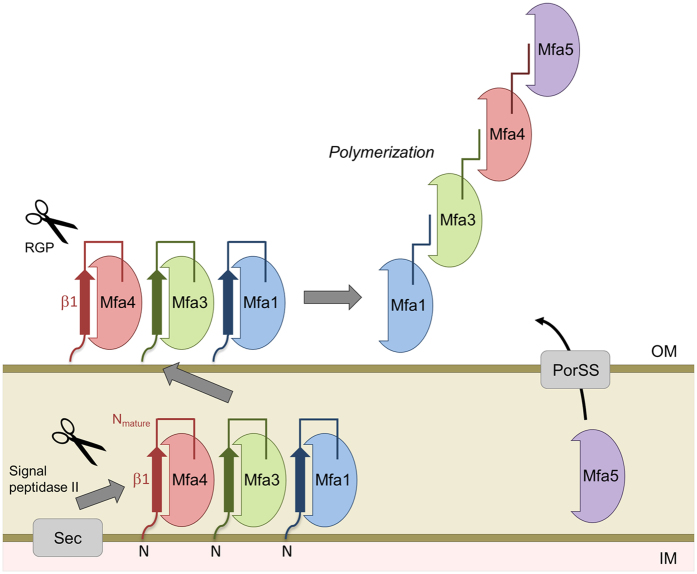
Proposed polymerization model of Mfa1 fimbria based on a donor strand complementation mechanism where the N_mature_ strand is inserted into a neighboring Mfa protein. The fimbrial proteins are transported to the inner membrane via the Sec system and to the outer membrane via the Lol pathway (Mfa1, Mfa3 and Mfa4). The accessory protein Mfa5 is transported to the outer membrane via the type IX secretion system.

So what is the function of the N-terminal extension? We hypothesize that it functions as an isogenous chaperone, filling sheet 1 before the polymerization reaction is performed. After the opening of the β1β2-loop by RGP, the β1-strand can be displaced by a β-strand originating from another fimbrial subunit, using a donor-strand complementation mechanism. If this is the mechanism, the donated strand must presumably originate from either the N- or C-terminus of another fimbrial subunit. If the polymerization mechanism in *P. gingivalis* is similar to that in *E. coli*, the N-terminus is the more likely candidate. We propose a model where the overhang formed by the remaining part of the cut β1β2-loop, from the protease site to the next β-strand (N_mature_), functions as a donor strand that can be incorporated into the next fimbrial subunit as the fimbria is polymerized (Fig. 7). In Mfa4 this stretch is 12 residues long, 14 in FimA, 15 in 4gpv and 16 in 3jfr (Fig. 5). These stretches are long enough to form a β-strand of the same length as, for example, the donor strand in the Caf1 protein of *Y. pestis*^24^ or the CS6 colonization factor of *E. coli*^26^. Since Mfa1 forms the major part of the fimbria, one can expect that a single Mfa1 protein donates a β-strand, either primarily to a neighbouring Mfa1, or secondarily to Mfa3. Mfa2 is proposed to control the growth and length of the polymer and is not identified as being part of the Mfa fimbria itself^9^. Recent studies have shown that Mfa3 is required for incorporation of Mfa4 and Mfa5 into the growing fimbria^12^; it is conceivable that the N_mature_ of Mfa3, which starts at Ala44, displaces the β1a strand of Mfa4 upon polymerization, thus completing the fold of the N-terminal domain. Further biochemical, biophysical and structural studies are necessary to confirm these hypotheses regarding the mechanisms underlying donor strand complementation and fimbrial assembly in *P. gingivalis*.

In addition to studying the role of the N-terminal extension, we also investigated the importance of the gingipain cleavage site in the β1β2 loop for obtaining a mature form of the Mfa4 protein that can be incorporated into the Mfa1 fimbria. The loop is typically cleaved at Arg53 by RGP. Nevertheless, cleavage still occurs after the exchange of the arginine for lysine or alanine. In the *fimA* null mutant *P. gingivalis* strain JI-1 Mfa4R53K mutant, the cleavage product was of the same size as the cleaved, wild-type protein, and we speculate that the lysine-specific gingipain, KGP, is responsible for the digestion. In the R53A mutant, the cleavage product was of slightly higher molecular weight and cleavage was confirmed to occur at Arg50. This indicates that either the position of the cleavage is most important as residue 53 is recognized independently of if an arginine or a lysine is present there, *or* that the RGP trimming can be performed stepwise, first at position 50 followed by 53 in the wild type protein. In the R50A/R53A double mutant, cleavage occurred at the nearest N-terminal lysine residue, Lys44, indicating that KGP can function as an alternative trimming enzyme when RGP sites are lacking. This was further confirmed upon analysis of the whole cell lysate from the RGP deficient mutant: Mfa4 protein in this mutant had the same molecular mass as it did in the R50A/R53A mutant. Furthermore, we demonstrated that Mfa4 is successfully incorporated into the mature fimbria independently of the final cleavage site. We also want to emphasize that no cleavage occurred downstream of Arg53, for example at Arg61; the N_mature_ was always left intact.

The structural and functional characterization of Mfa4 and related fimbrial proteins may aid in the development of anti-bacterial substances that target the polymerization machinery, or block the binding of the individual proteins. Therefore, future efforts will focus on the further characterization of the polymerization mechanism and the identification and characterization of the interaction partners of the individual fimbrial proteins.

## Materials and Methods

### Cloning

The *mfa4* gene (GenBank accession code AP009380) was cloned in two forms from genomic DNA of *P. gingivalis* strain ATCC 33277. The mature form of the protein encodes residues 54–333 and the precursor form residues 26–333. For primers see Table S1. The PCR products were digested with *Nco*I and *Acc65*I and ligated into the equivalent sites of the pET-His1a expression vector (kindly provided by G. Stier, EMBL, Germany). The final constructs encoded His6-PMSDYDIPTTENLYFQGAM before the start of the respective constructs.

### Overexpression and purification

The protein was overexpressed in *E. coli* BL21 (DE3) in auto induction media (Luria Broth containing 0.001% glucose, 0.02% lactose and 0.02% glycerol) supplemented with 50 μg mL^−1^ kanamycin. After 3 hours at 37 °C the temperature was lowered to 22 °C and the culture was grown for 16 hours. Cells were harvested by centrifugation at 5300 × *g* and the pellets were frozen at −80 °C. Cell pellets were resuspended in 50 m*M* Tris-HCl pH 8.0, 0.3 *M* NaCl and 10 m*M* imidazole supplemented with 1% triton-X 100 and an EDTA-free protease inhibitor cocktail (Roche). The suspension was lysed on ice by sonication and cellular debris was removed by centrifugation at 39000 × *g* for 35 min. The supernatant was loaded onto a column packed with Ni-NTA agarose (Qiagen). The proteins bound on the column were washed with 50 m*M* Tris-HCl pH 7.5, 0.3 *M* NaCl and 20 m*M* imidazole and eluted with 50 m*M* TrisHCl pH 7.5, 0.3 *M* NaCl and 0.3 *M* imidazole. The buffer was exchanged to 50 m*M* Tris-HCl pH 7.5, 0.2 *M* NaCl and 0.5 m*M* EDTA for further purification by size-exclusion chromatography (HiLoad^TM^ 16/60 Superdex^TM^ 200 prep-grade column, Amersham Biosciences). Fractions containing the target protein were concentrated in 25 m*M* Tris-HCl pH 7.5 using an Amicon Ultra centrifugal filter device (Millipore). The protein purity was assessed by SDS-PAGE.

To express selenomethionine-substituted Mfa4, cells were grown in M9 media supplemented with glucose at 37 °C. At an optical density of ~0.4 at 600 nm, lysine, threonine, phenylalanine at 100 mg L^−1^, leucine, isoleucine, valine, proline and selenomethionine at 50 mg L^−1^ were added to downregulate the synthesis of methionine^27^. The selenomethionine-labelled protein was purified as described above with the exception in all steps.

### Crystallization and data collection

Initial crystallization trials were performed with 15 mg mL^−1^ protein by the sitting-drop vapor-diffusion method in a 96-well MRC-crystallization plate (Molecular Dimensions) using a Mosquito (TTP Labtech) pipetting robot. In addition, trials were performed in parallel in which 1% (w/w) chymotrypsin was added to the protein solution immediately before crystallization screening. This *in situ* proteolysis with chymotrypsin was performed as a means to trim off flexible parts to facilitate crystal growth^28^. Droplets of 0.1 μL protein solution were mixed with an equal volume of reservoir solution using screens from Hampton Research and Molecular Dimensions. Crystals of α-chymotrypsin-treated protein were obtained from 5% (w/v) polyglutamic acid (PGA), 20% (w/v) PEG 4000 and 0.1 *M* Tris-HCl, pH 8.0 at 18 °C. Crystals were soaked for 30 seconds in this mother liquor solution supplemented with 20% (v/v) glycerol before they were flash cooled in liquid nitrogen and stored until data collection.

Diffraction data to 1.9 Å resolution from the selenomethionine-substituted crystals were collected on a Pilatus 6M-F detector at beam ID23-1 at the European Synchrotron Radiation Facility, Grenoble, France. Diffraction images were processed with XDS^29^ and scaled with SCALA from the CCP4 program suit^30^. Relevant processing statistics are summarized in Table 1.

### Structure determination and refinement

The structure of selenomethionine-labelled Mfa4 was solved with SAD-phasing using AutoRickshaw^31^. Density modification and automatic model building were performed using AutoRickshaw and ArpWarp^32^ and resulted in a readily interpretable map. For refinement, 5% of the reflections were removed for the calculation of R_free_. The model was further built using rounds of manual building in COOT^33^ and refined using phenix.refine^34^. Two molecules were found in the asymmetric unit, which corresponds to a Matthews number of 2.1 Å ^3^Da^−1^ (40.1% solvent)^35^.

In the last rounds of refinement, translational-libration-screw^36^ refinement was used, treating each molecule as an individual TLS group. Hydrogen atoms were included and refined in the final model. The quality of the model was analyzed with MolProbity in PHENIX^37^. Crystallographic statistics are summarized in Table 1. Figures were drawn with CCP4MG^38^. The X-ray coordinates and structure factors have been deposited in the Protein Data Bank under accession code 5dhm.

### Generation of the point mutants

The Mfa4 R53A or R53K mutants in *P. gingivalis* JI-1 (Δ*fimA*) were generated using the PCR-based overlap extension method^11^,^12^. The primers and their annealing sites are shown in Supplementary Table S1 and Figure S3, respectively. The *tetQ* gene was amplified with primers TetQF and TetQR to generate a 2.2-kbp product from pT-COW^39^. For construction of the R53A or R53K-point mutation cassettes, the *mfa4* sequence was amplified with primers Mfa4WTFU, Mfa4R53AFU or Mfa4R53KU, and Mfa4RU that have homology to the 5′ end of the *tetQ* fragment. The flanking sequence downstream of *mfa4* was amplified with Mfa4FD and Mfa4RD that have homology to the 3′ end of the *tetQ* fragment. The *tetQ*, *mfa4* fragment with the point mutation on R53A or R53K, and *mfa4*-downstream fragments, were used as templates for overlap extension PCR to generate the point mutation cassettes.

The Mfa4R50A/R53A double mutant was generated in a similar manner. The primers and their annealing sites are shown in Supplementary Table S1 and Figure S4, respectively. In brief, for construction of the R50A/R53A-point mutation cassettes, the flanking *mfa4* sequence including *tetQ* of *P. gingivalis* Mfa4R53A was amplified with primers Mfa4R50AR53AF and Mfa4RD. The upstream of *mfa4* gene was amplified with primers Mfa4FU and Mfa4R50AR53AR that have homology to the 3′ end of the upstream of *mfa4* fragment. The upstream and *mfa4* fragments with the point mutation on R53A/R50A were used as templates for overlap extension PCR to generate the point mutation cassette.

Each cassette created was ligated into pCR-Blunt II-TOPO, and the resulting recombinant plasmids were transformed into competent cells of *E. coli* TOP10 according to the manufacturer’s directions (Thermo Fisher Scientific Inc.).

Electroporation of *P. gingivalis* was performed as described previously^12^. The plasmid constructs were linearized by digestion with endonuclease *Xba*I and introduced into electrocompetent cells of *P. gingivalis* JI-1. After 16 h of anaerobic incubation in trypticase soy broth supplemented with 0.25% yeast extract, 2.5 μg mL^−1^ hemin, 5 μg mL^−1^ menadione, and 0.1 μg mL^−1^ DTT, the pulsed cells were plated on Brucella HK agar (Kyokuto Pharmaceutical Industrial) supplemented with 5% laked rabbit blood, 2.5 μg mL^−1^ hemin, 5 μg mL^−1^ menadione, 0.1 μg mL^−1^ DTT, and 1 μg mL^−1^ tetracycline, and the plates were incubated anaerobically at 37 °C for 7 days. Possible transformants were verified by PCR and DNA sequencing.

### Sample preparation, SDS-PAGE and immunoblotting

Preparation of the bacterial whole-cell lysate, SDS-PAGE and immunoblotting were performed as described previously^12^. The gels were stained with Coomassie brilliant blue R-250 (CBB). Antigen-specific antisera against Mfa4 protein were used as primary antibodies in immunoblotting.

### Purification of Mfa1 fimbriae

Mfa1 fimbriae were purified as described previously^9^. Briefly, cells were lysed by French press, the soluble fraction cleared by ultracentrifugation and precipitated with ammonium sulfate at 50% saturation. Mfa1 fimbriae were purified by ion exchange and size exclusion chromatography. Purity and identity were verified by SDS-PAGE and mass spectrometry.

### N-terminal sequencing

The purified fimbriae from *P. gingivalis* JI-1 Mfa4R53A and Mfa4R50A/R53A, separated on SDS-PAGE, were electrophoretically transferred to PVDF membranes and stained with Coommassie Brilliant Blue. The Mfa4 bands were excised and analyzed by N-terminal sequencing on an ABI 477 A automatic peptide sequence analyzer at the Center for Instrumental Analysis, Hokkaido University, Japan.

## Additional Information

**How to cite this article**: Kloppsteck, P. *et al*. Structure of the fimbrial protein Mfa4 from *Porphyromonas gingivalis* in its precursor form: implications for a donor-strand complementation mechanism. *Sci. Rep*. **6**, 22945; doi: 10.1038/srep22945 (2016).

## Supplementary Material

Supplementary Information

## Figures and Tables

**Figure 1:**
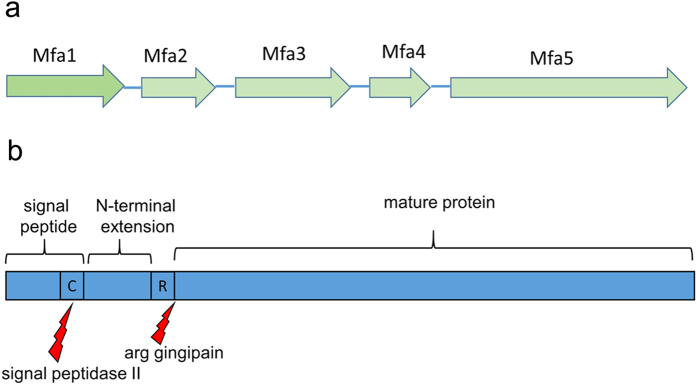
Schematic diagrams of the Mfa1 gene cluster and the Mfa4 protein. (**a**) The five genes that encode the Mfa1 fimbrial proteins; Mfa1 forms the shaft of the protein, Mfa2 regulates the length of the fimbriae and Mfa3-5 are tip proteins. (**b**) Mfa4 starts with an 18 aa long signal sequence followed by a lipidated cysteine. The mature form of the protein starts at Asn54 after gingipain cleavage at Arg53. Residues 19–53 are referred to as the N-terminal extension.

**Table 1:** Data collection, refinement and model quality statistics for Mfa4.

	selenomethionine-substituted Mfa4_26–333_
Data processing	
Space group	P2_1_P2_1_P2_1_
Cell dimensions *a*, *b*, *c* (Å)	54.68, 84.54, 138.36
Resolution (Å)	1.90
Highest resolution shell (Å)	2.00–1.90
Total observations	327588
Unique reflections	51290
<*I*/σ (*I*)^a^>	16.0 (3.4)
*R*_pim_^a^(%)	2.7 (20.6)
Completeness (%)^a^	99.5 (97.8)
Overall redundancy^a^	6.4 (6.2)
Refinement	
No. of reflections in working set	48676
No. of reflections in test set	2614
*R*_work_/*R*_free_ (%)	18.4/22.9
RMSD from ideal	
Bond lengths (Å)	0.008
Bond angles (°)	1.12
Wilson B-factor (Å^2^) Average *B*-factors (Å^2^)	32.1
Protein (A, B)	42, 9, 55, 7
Water	37.6
Ramachandran plot (%)	
Favored, allowed	97.7, 0.5

^a^Values in parentheses indicate statistics for the highest resolution shell.

**Table 2:** Structurally related proteins obtained by DALI.

PDB	Z-score	Rmsd	Lali	nres	% identity	Description	Organism	lipidated cystein	Suggested gingipain site	LipoP score
Mfa4 5dhm	–	–	–	–	–	Mfa4	*Porphyromonas gingivalis* (*ATCC33277*)	Cys 19	Arg53*	11.4
4gpv	15.9	3.1	221	327	19	putative cell adhesion protein	*Bacteroides eggerthii*	Cys19	Arg53	18.9
3r4r	14.9	3.3	206	274	15	hypothetical fimbrial assembly	*Parabacteroides distasonis*	*Cys21*	*Lys50*	13.1
4jg5	14.5	3.1	216	336	18	putative cell adhesion protein	*Parabacteroides distasonis*	*Cys20*	*Lys47*	13.1
3liu	14.4	2.8	214	372	18	putative cell adhesion protein	*Parabacteroides distasonis*	*Cys19*	*Lys43*	19.2
3up6	14.2	3.3	227	324	19	hypothetical protein	*Bacteroides ovatus*	*Cys20*	*Lys54*	6.2
4jrf	13.8	3.2	218	479	18	putative cell adhesion protein	*Bacteroides ovatus*	*Cys20*	*Arg55*	17.9
4q98	13.3	3.1	207	357	16	FimA	*Porphyromonas gingivalis* (*W83*)	*Cys19*	Arg45*	20.5

*Confirmed cleavage site.

## References

[b1] Socransky S. S. & Haffajee A. D. Periodontal microbial ecology. Periodontol 2000 38, 135–87 (2005). 15853940 10.1111/j.1600-0757.2005.00107.x

[b2] Atanasova K. R. & Yilmaz O. Looking in the Porphyromonas gingivalis cabinet of curiosities: the microbium, the host and cancer association. Mol Oral Microbiol 29, 55–66 (2014). 24506890 10.1111/omi.12047 PMC3949145

[b3] Michaud D. S. . Plasma antibodies to oral bacteria and risk of pancreatic cancer in a large European prospective cohort study. Gut 62, 1764–70 (2013). 22990306 10.1136/gutjnl-2012-303006 PMC3815505

[b4] Ishikawa M. . Oral Porphyromonas gingivalis translocates to the liver and regulates hepatic glycogen synthesis through the Akt/GSK-3beta signaling pathway. Biochim Biophys Acta 1832, 2035–43 (2013). 23899607 10.1016/j.bbadis.2013.07.012

[b5] Lamont R. J. & Jenkinson H. F. Life below the gum line: pathogenic mechanisms of Porphyromonas gingivalis. Microbiology & Molecular Biology Reviews 62, 1244–63 (1998). 9841671 10.1128/mmbr.62.4.1244-1263.1998 PMC98945

[b6] Sato K. . Identification of Porphyromonas gingivalis proteins secreted by the Por secretion system. FEMS Microbiol Lett 338, 68–76 (2013). 23075153 10.1111/1574-6968.12028

[b7] Amano A. Molecular interaction of Porphyromonas gingivalis with host cells: implication for the microbial pathogenesis of periodontal disease. J Periodontol 74, 90–6 (2003). 12593602 10.1902/jop.2003.74.1.90

[b8] Yoshimura F., Murakami Y., Nishikawa K., Hasegawa Y. & Kawaminami S. Surface components of Porphyromonas gingivalis. J Periodontal Res 44, 1–12 (2009). 18973529 10.1111/j.1600-0765.2008.01135.x

[b9] Hasegawa Y. . Anchoring and length regulation of Porphyromonas gingivalis Mfa1 fimbriae by the downstream gene product Mfa2. Microbiology 155, 3333–47 (2009). 19589838 10.1099/mic.0.028928-0 PMC2810400

[b10] Nagano K., Hasegawa Y., Murakami Y., Nishiyama S. & Yoshimura F. FimB regulates FimA fimbriation in Porphyromonas gingivalis. J Dent Res 89, 903–8 (2010). 20530728 10.1177/0022034510370089

[b11] Hasegawa Y. & Murakami Y. Porphyromonas gingivalis fimbriae: Recent developments describing the function and localization of mfa1 gene cluster proteins. Journal of Oral Biosciences 56, 86–90 (2014).

[b12] Hasegawa Y. . Localization and function of the accessory protein Mfa3 in Porphyromonas gingivalis Mfa1 fimbriae. Mol Oral Microbiol 28, 467–80 (2013). 24118823 10.1111/omi.12040

[b13] Gerlach R. G. & Hensel M. Protein secretion systems and adhesins: the molecular armory of Gram-negative pathogens. Int J Med Microbiol 297, 401–15 (2007). 17482513 10.1016/j.ijmm.2007.03.017

[b14] Thanassi D. G., Saulino E. T. & Hultgren S. J. The chaperone/usher pathway: a major terminal branch of the general secretory pathway. Curr Opin Microbiol 1, 223–31 (1998). 10066482 10.1016/s1369-5274(98)80015-5

[b15] Kadowaki T. . Arg-gingipain acts as a major processing enzyme for various cell surface proteins in Porphyromonas gingivalis. J Biol Chem 273, 29072–6 (1998). 9786913 10.1074/jbc.273.44.29072

[b16] Juncker A. S. . Prediction of lipoprotein signal peptides in Gram-negative bacteria. Protein Sci 12, 1652–62 (2003). 12876315 10.1110/ps.0303703 PMC2323952

[b17] Okuda S. & Tokuda H. Lipoprotein sorting in bacteria. Annu Rev Microbiol 65, 239–59 (2011). 21663440 10.1146/annurev-micro-090110-102859

[b18] McNicholas S., Potterton E., Wilson K. S. & Noble M. E. Presenting your structures: the CCP4mg molecular-graphics software. Acta Crystallogr D Biol Crystallogr 67, 386–94 (2011). 21460457 10.1107/S0907444911007281 PMC3069754

[b19] Holm L. & Rosenstrom P. Dali server: conservation mapping in 3D. Nucleic Acids Res 38, W545–9 (2010). 20457744 10.1093/nar/gkq366 PMC2896194

[b20] Shoji M. . The major structural components of two cell surface filaments of Porphyromonas gingivalis are matured through lipoprotein precursors. Mol Microbiol 52, 1513–25 (2004). 15165251 10.1111/j.1365-2958.2004.04105.x

[b21] Hussain M., Ichihara S. & Mizushima S. Mechanism of signal peptide cleavage in the biosynthesis of the major lipoprotein of the Escherichia coli outer membrane. J Biol Chem 257, 5177–82 (1982). 7040395

[b22] Nakayama K., Kadowaki T., Okamoto K. & Yamamoto K. Construction and characterization of arginine-specific cysteine proteinase (Arg-gingipain)-deficient mutants of Porphyromonas gingivalis. Evidence for significant contribution of Arg-gingipain to virulence. J Biol Chem 270, 23619–26 (1995). 7559528 10.1074/jbc.270.40.23619

[b23] Choudhury D. . X-ray structure of the FimC-FimH chaperone-adhesin complex from uropathogenic Escherichia coli. Science 285, 1061–6 (1999). 10446051 10.1126/science.285.5430.1061

[b24] Zavialov A. V. . Resolving the energy paradox of chaperone/usher-mediated fibre assembly. Biochem J 389, 685–94 (2005). 15799718 10.1042/BJ20050426 PMC1180718

[b25] Sauer F. G., Pinkner J. S., Waksman G. & Hultgren S. J. Chaperone priming of pilus subunits facilitates a topological transition that drives fiber formation. Cell 111, 543–51 (2002). 12437927 10.1016/s0092-8674(02)01050-4

[b26] Roy S. P. . Crystal structure of enterotoxigenic Escherichia coli colonization factor CS6 reveals a novel type of functional assembly. Mol Microbiol 86, 1100–15 (2012). 23046340 10.1111/mmi.12044

[b27] Van Duyne G. D., Standaert R. F., Karplus P. A., Schreiber S. L. & Clardy J. Atomic structures of the human immunophilin FKBP-12 complexes with FK506 and rapamycin. Journal of Molecular Biology 229, 105–24 (1993). 7678431 10.1006/jmbi.1993.1012

[b28] Dong A. . *In situ* proteolysis for protein crystallization and structure determination. Nat Methods 4, 1019–21 (2007). 17982461 10.1038/nmeth1118 PMC3366506

[b29] Kabsch W. Xds. Acta Crystallogr D Biol Crystallogr 66, 125–32 (2010). 20124692 10.1107/S0907444909047337 PMC2815665

[b30] Collaborative Computational Project, N. The CCP4 suite: programs for protein crystallography. Acta Crystallogr D Biol Crystallogr 50, 760–3 (1994). 15299374 10.1107/S0907444994003112

[b31] Panjikar S., Parthasarathy V., Lamzin V. S., Weiss M. S. & Tucker P. A. Auto-Rickshaw: an automated crystal structure determination platform as an efficient tool for the validation of an X-ray diffraction experiment. Acta Crystallogr D Biol Crystallogr 61, 449–57 (2005). 15805600 10.1107/S0907444905001307

[b32] Langer G., Cohen S. X., Lamzin V. S. & Perrakis A. Automated macromolecular model building for X-ray crystallography using ARP/wARP version 7. Nat Protoc 3, 1171–9 (2008). 18600222 10.1038/nprot.2008.91 PMC2582149

[b33] Emsley P., Lohkamp B., Scott W. G. & Cowtan K. Features and development of Coot. Acta Crystallogr D Biol Crystallogr 66, 486–501 (2010). 20383002 10.1107/S0907444910007493 PMC2852313

[b34] Afonine P. V. . Towards automated crystallographic structure refinement with phenix.refine. Acta Crystallogr D Biol Crystallogr 68, 352–67 (2012). 22505256 10.1107/S0907444912001308 PMC3322595

[b35] Matthews B. W. Solvent content of protein crystals. Journal of Molecular Biology 33, 491–7 (1968). 5700707 10.1016/0022-2836(68)90205-2

[b36] Winn M. D., Isupov M. N. & Murshudov G. N. Use of TLS parameters to model anisotropic displacements in macromolecular refinement. Acta Crystallographica Section D-Biological Crystallography 57, 122–33 (2001). 10.1107/s0907444900014736 11134934

[b37] Chen V. B. . MolProbity: all-atom structure validation for macromolecular crystallography. Acta Crystallogr D Biol Crystallogr 66, 12–21 (2010). 20057044 10.1107/S0907444909042073 PMC2803126

[b38] Potterton L. . Developments in the CCP4 molecular-graphics project. Acta Crystallographica Section D-Biological Crystallography 60, 2288–94 (2004). 10.1107/S0907444904023716 15572783

[b39] Gardner R. G., Russell J. B., Wilson D. B., Wang G. R. & Shoemaker N. B. Use of a modified Bacteroides-Prevotella shuttle vector to transfer a reconstructed beta-1,4-D-endoglucanase gene into Bacteroides uniformis and Prevotella ruminicola B(1)4. Appl Environ Microbiol 62, 196–202 (1996). 8572695 10.1128/aem.62.1.196-202.1996 PMC167786

[b40] Ikai R. . Mfa4, an Accessory Protein of Mfa1 Fimbriae, Modulates Fimbrial Biogenesis, Cell Auto-Aggregation, and Biofilm Formation in Porphyromonas gingivalis. Plos One 10, e0139454 (2015). 26437277 10.1371/journal.pone.0139454 PMC4593637

