# Supplementary material for: Structure of the fimbrial protein Mfa4 from Porphyromonas gingivalis in its precursor form: implications for a donor-strand complementation mechanism
Source: Sci Rep. 2016 Mar 14;6:22945. doi: 10.1038/srep22945 (PMC4789730; doi:10.1038/srep22945)
Supplement: Supplementary Information [file srep22945-s1.pdf]

Supplementary information

Structure of the fimbrial protein Mfa4 from *Porphyromonas gingivalis* in its precursor form: implications for a donor-strand complementation mechanism

Patrik Kloppsteck[1], Michael Hall[1], Yoshiaki Hasegawa[2] and Karina Persson[1*]

[1]Department of Chemistry, Umeå University, Umeå, Sweden, SE-901 85

[2]Department of Microbiology, School of Dentistry, Aichi Gakuin University, 1-100 Kusumoto-cho, Chikusa-ku, Nagoya, Aichi 464-8650, Japan

[*]Correspondence to Karina Persson, Department of Chemistry, Umeå University, SE-90185 Umeå, Sweden, email: karina.persson@chem.umu.se

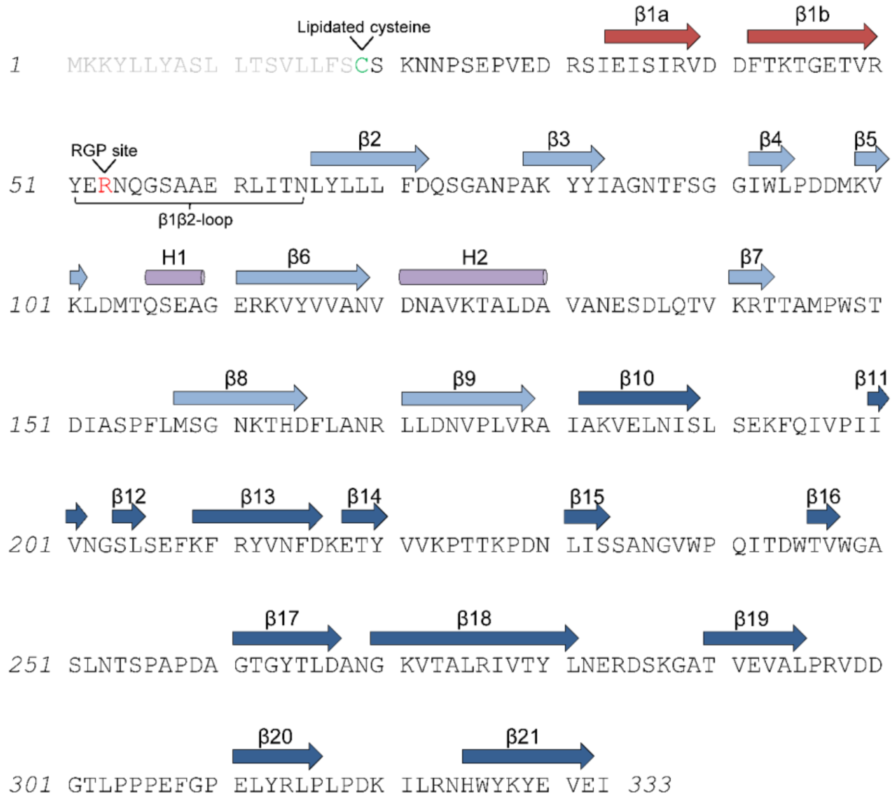

Supplementary figure S1. Sequence and secondary structure of Mfa4. Helices are depicted as cylinders and strands as arrows. The lipidated cysteine, Cys19, and the RGP recognition site, Arg53 are indicated in green and red, respectively

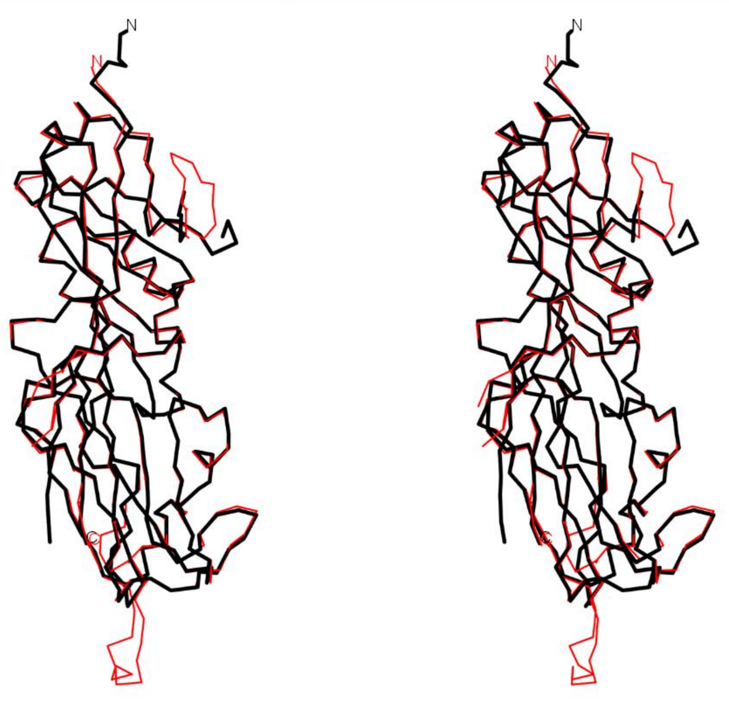

Supplementary figure S2. Comparison of Mfa4 and PG0181. Superposition of Mfa4 from strain ATCC 33277 and PG0181 from strain W83 (PDB code 4rdb). Mfa4 is colored red and PG0181 black. The figure is shown in stereo.

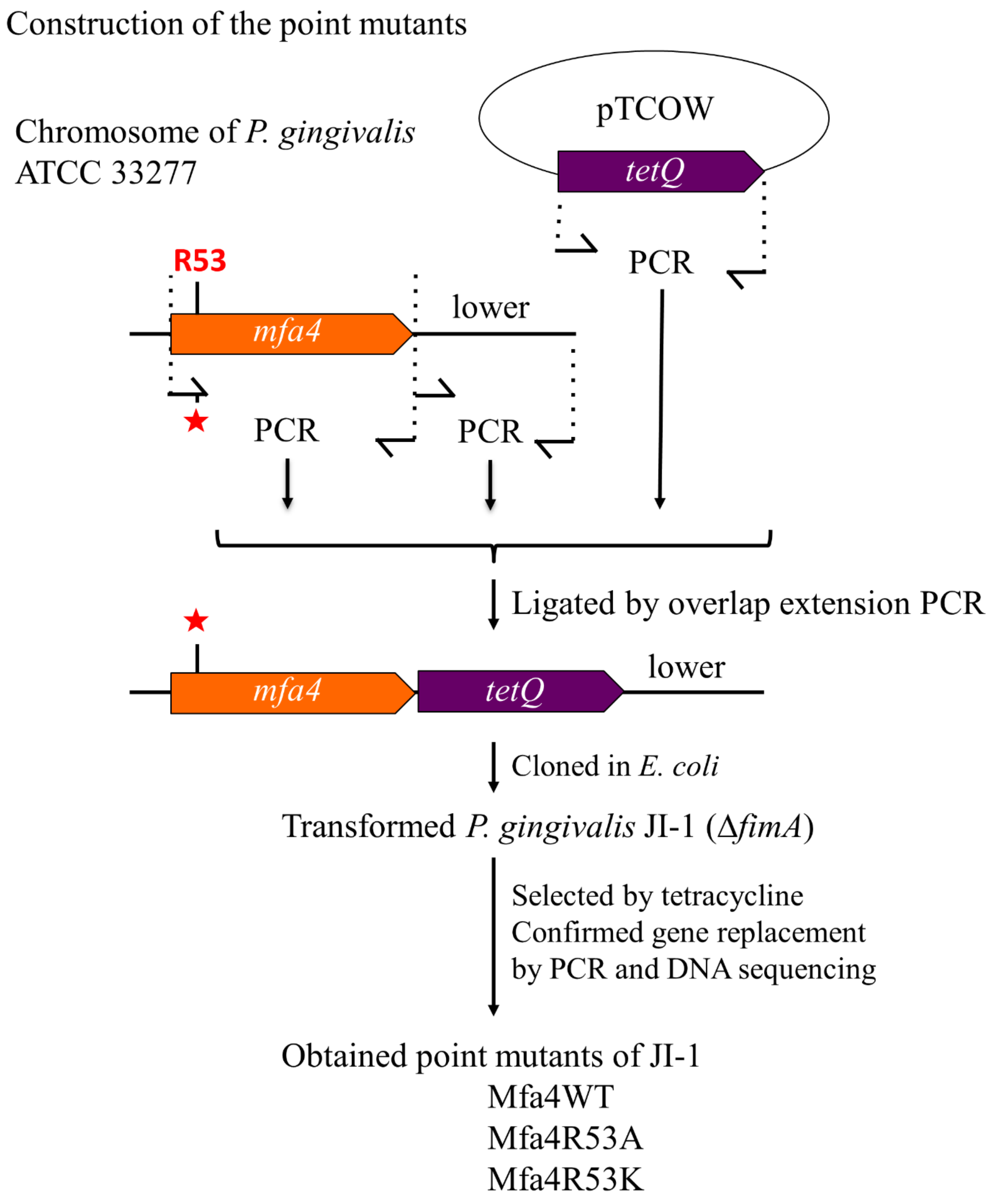

Supplementary figure S3. Construction of point mutants in *mfa4* of *P. gingivalis*. Small arrows show the primers. R, arginine residue; star, alanine or lysine residues.

Construction of the double point mutant

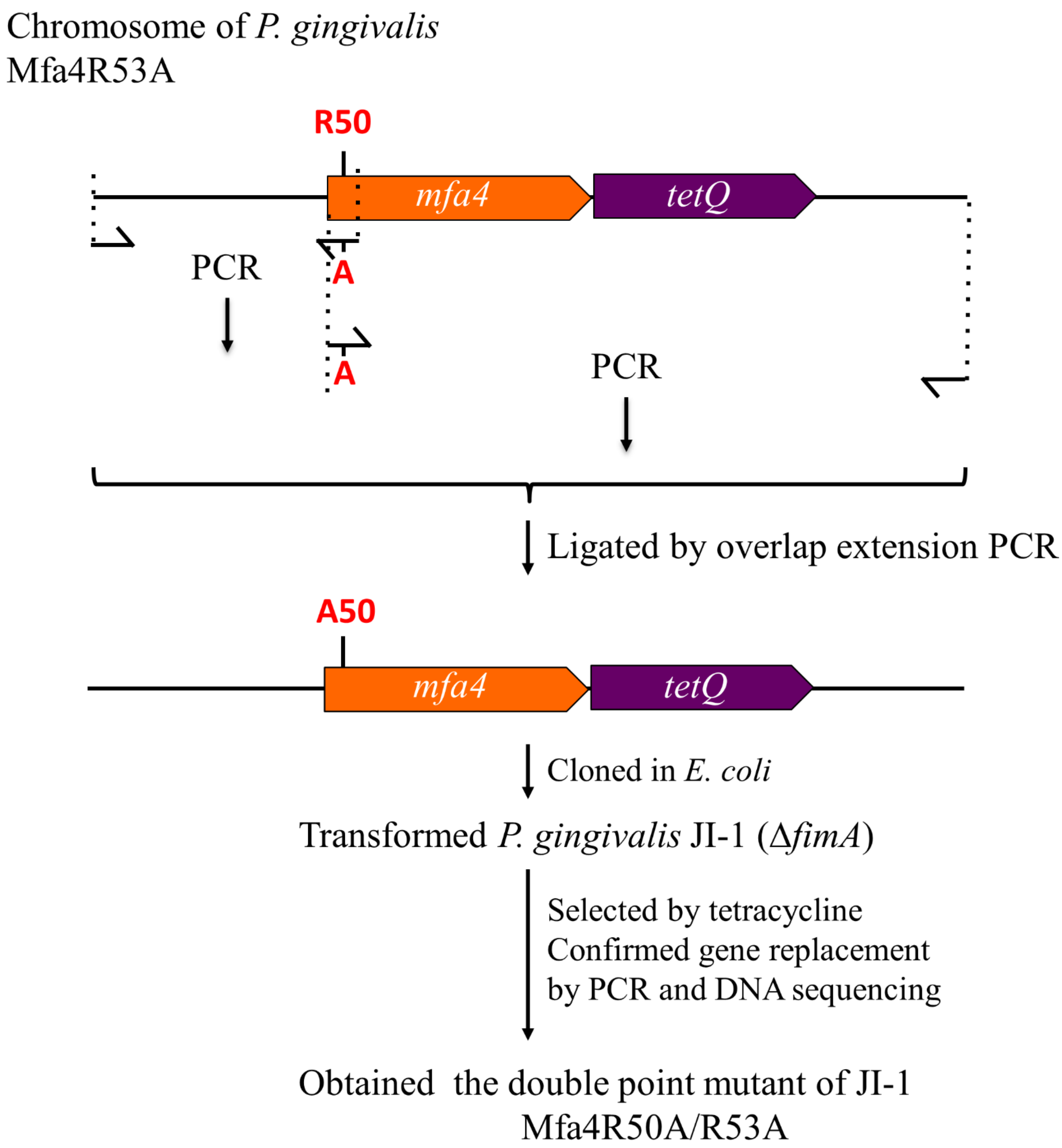

Supplementary figure S4. Construction of the double point mutant in *mfa4* of *P. gingivalis*.

Small arrows show the primers. R, arginine residue; A, alanine residue.

Table S1 Primers used for generation of expression constructs and point mutants.

Primer	Sequence (5’-3’)	Description of underline
Mfa4mature	TGATACCATGGCTAATCAAGGAAGTGCTGCC	*NcoI* site
Mfa4precursor	TTTTTCCATGGAGCCGGTGGAAGACAGA	*NcoI* site
Mfa4 C-term	TTTTTGGTACCTCAAATCTCGACTTCGTACTT	*Acc65I* site
TetQF	ACTTACCAGTTGAACCTACGTTTCC	
TetQR	CTTATAGAAATTTCCTGACCGCTCC	
Mfa4WTFU	GACAGTACGCTATGAAAGGAATCAAGGAAG TGCTGCCGAAAGGCTCATTACCAATC	
Mfa4R53AFU	GACAGTACGCTATGAAGCGAATCAAGGAAG TGCTGCCGAAAGGCTCATTACCAATC	Ala mutation
Mfa4R53KFU	GACAGTACGCTATGAAAAGAATCAAGGAAG TGCTGCCGAAAGGCTCATTACCAATC	Lys mutation
Mfa4RU	GGAAACGTAGGTTCAACTGGTAAGTTCAAA TCTCGACTTCGTACTTGTACCAATGATTG	Overlapping region of 5′ end of *tetQ*
Mfa4FD	GGAGCGGTCAGGAAATTTCTATAAGTATTTT TTAGGTCTTGTT TGATATTGTG GAAGAG	Overlapping region of 3′ end of *tetQ*
Mfa4RD	CGAAGACTGGTCGATAACCATCACCACATC	
Mfa4R50AR53 AF	GACAGTAGCCTATGAAGCGAATCAAGGAAG TGCTGCCGAAAGGCTCATTACCAATC	Ala mutations and overlapping region of 3′ end of upstream fragment
Mfa4FU	GAGTAGGTGCCACGAACCAATGG	
Mfa4R50AR53 AR	GATTCGCTTCATAGGCTACTGTC	Ala mutations
